# Odonto-Chirurgical Society

**Published:** 1888-04

**Authors:** 


AMERICAN JOURNAL

—OF—

DENTAL SCIENCE.

VOL. XXI. THIRD SERIES—APRIL, 1888. No. 12.

ARTICLE I.

### ODONTO-CHIRURGICAL SOCIETY.

### SOME REMARKS WITH REGARD TO ARTIFICIAL CROWNS.

BY DR. WILLIAMSON.

#### METHODS OF ATTACHMENT.

This part of the subject might easily be developed into tremendous proportions, as dental ingenuity would seem to have been exercised to its utmost in the endeavour to devise an ideal method by which a crown may be attached to a root.

There are probably some seventy or eighty methods, or variations of methods, and in each one of these there is some objectionable feature or features; the reason being, as in many other things, the difficulty of combining all the desirable qualities, which may be classified as follows:—

1. Natural appearance of crown.

2. Perfect fixity and strength of attachment of crown to root.

3. Preservation of root from decay.

To accomplish these principal ends, varieties of two methods have been employed, (1) attachment by means of a pin in the root; (2) attachment by means of a collar or band encircling and fitting the end of the root, generally combined with the use of a pin.

I will not enter into the various details, but merely make a few remarks in regard to those methods that I have had some experience of, and, to do this, the best way will be to make a division of the teeth into two classes—(1) Incisors and Canines; (2) Bicuspids and Molars.

1. *Incisors and Canines.*—This is the class that we are most frequently called on to treat in the way of artificial crowns, and where there is most reason for the operation, because towards the front of the mouth the alternatives of a suction or grasp plate are both objectionable, the former from its bulk, and the latter from the probable injury to the teeth in the case of a careless person, and from the difficulty of concealment of the fastenings in some cases. The loss of a front tooth is a very potent way of bringing the patient to the dentist, especially with ladies, because of course the vacancy is so unsightly, and for this reason some little trouble should be exercised in order to obtain a substitute of natural shade and colour. In many cases this is a most difficult matter, and to me sometimes a worrying one. Even with a large stock to select from, there are various causes which increase the difficulty, as, for example, in the case of a lateral, where there is the different shade of the adjoining teeth, the light shade of the central and the darker one of the canine, the lateral being selected approximately more to the central. Again, where teeth are discoloured by approximal filling, it is difficult to make an artificial tooth appear natural beside it, except by colouring it on the same side to match the stained one. This is one of the most useful ways in which the method of tinting teeth, lately introduced, can be employed, affording a means of gradation from the natural to the artificial, not obtainable otherwise, and I have employed it in several cases with advantage. An extra deep shade of grey or blue on one side of the tooth, and shaded off towards the centre, is a very effective means of concealing the transition. I had a case lately where a central incisor had to be replaced, the other one being stained a peculiar shade of yellow, as the result of an old injury. There was one tooth among those I had which was a fair match, but, being too short, I sent to a London house for a selection to match this colour, A score was sent, but none that could be called very near it, so the only resort was the painting method, which, after a number of trials, yielded a very fair approximation to the colour desired. There is one caution in regard to those painted teeth, and that is, they should be protected from the action of acid when the metal portion is boiled in it. Some of the colours seem to be affected by acid more than others. As to wear in the mouth, as far as I have seen, they stand very well. The fact of the not infrequent difficulty of selection of colour and size together is often so great, even with the assistance of the painting process, as to be, in my opinion, a fatal objection to the use in the front of the mouth of special crowns, such as the Bonwill, Howe, Weston, Logan, and others. Without sufficient compensating advantages, it is not worth while for any ordinary practitioner to keep a large number of any of these forms in order that he may find a suitable one for any particular case, for even then he may be at a loss for one; for instance, I noticed in the report of a New York clinic, held the other day in a dental depot, that the operator who was to set one of these special crowns could not proceed because he could not obtain one adapted for the case in hand. A plain tooth, then, is, generally speaking, the best form to employ, as it gives the greatest opportunity of fulfilling the primary object of a natural appearence, and if broken, can be readily replaced. As to the methods of attachment, I used at first the old method of a gold pin, with silk covered with varnish, and forced up into the canal. This method, although old-fashioned, is not to be despised in the case of a sound root, and not a few in this country still think it one of the best —an opinion which would seem to be confirmed by the cases one meets with where such a tooth crown has lasted for many years. Still, in spite of some favorable cases, I do not continue its practice, as I always felt a degree of uncertainty as to whether, when the tooth was actually in position, it was tight enough, and if there was rather too much silk on the pin then it would not go far enough, a state of things not easy to remedy.

On the Bonwill crowns being brought out, I tried a few of them in cases of the first class, but did not find them very satisfactory, there being several causes of failure. The first one I used was in the case of a young clergyman who had a badly decayed canine root. The crown was set with amalgam, the pin used being the strongest form, of an elongated triangular shape, and the operation was in every way satisfactory; but three months afterwards he came with the tooth broken off, the pin having given way right through the angle which is the thickest part. Another one, a lateral, lasted three years, but, in this case, the lateral pin being very small, the strength lay in the amalgam. A central lasted about the same time, and finally the tooth substance broke at the back part of the ring, the weak part in all those of a similar pattern, because the crown is more or less weakened according to the amount of filling required, and also in the case of a close bite, where it requires grinding on the lingual side. In regard to the Bonwill pins, it is but fair to say that those I used were of the first pattern. Since then they have been made without serrations and of greater strength.

My next experience was with the system best known as Balkwill's from his having devised a set of iustruments for the performance of the operation by means of a tooth soldered to a plate fitting the top of a root, and this again attached to a split platinum pin, which is forced up into a platinum tube screwed into the root canal. This is the method I have used most in cases of fresh sound roots, putting on first some low grade gutta-percha on the root side of the plate, gently warming it over the spirit lamp, and then pressing it up into place, and of course trimming off the surface with warm instruments. It is one of the few methods that permit of the removal of the crown entire, but at the same time the pin must not fit the tube too easily, otherwise it would soon loosen. The quality of removability has led to its employment in removable bridge work. What I find most troublesome is the preparation of the root for the screw tube, the tapping being a slow process, and apt to give the patient the idea of the preliminary stages of root extraction by means of the screw, owing to the tap not cutting readily enough not to prevent a series of partial rotations of the root along with it. However, if the root is healthy, no trouble seems to be caused by the strain imposed in this way. People in my part of the country seem to have a general idea that pivoted teeth are screwed in, a peculiar accent being given to the word, which seems to imply some notion of great pain accompanying the operation, and so, partly for this reason, although it is not really more disagreeable, I would prefer to avoid the tapping process, and I do not know that it is really necessary, as what will do probably as well is a tube, roughened on the outside by a bit or two of wire soldered on, first fixed in the root with a little oxyphosphate towards the apex, and then, say, the other half packed round with amalgam, the calibre of the root canal having been enlarged to allow of it being inserted with ease and certainty. Not only is this method applicable for sound roots, but especially for cases where there is a deficiency of the root substance at the sides, perhaps where the gum has had to be pressed out to expose the surface. The missing wall may be built up with amalgam retained by a groove on both sides, aided by the screw thread or roughened surface of the tube.

In cases where the root is very much hollowed and enlarged, as in those caused by the patient having worn an ordinary gold pin with plenty of silk on it, but where there is no deficiency at any of the sides, then I think a crown and pin set with oxyphosphate is the best treatment. The tooth is attached to a strong platinum pin, as long as the root will allow. To strengthen and give the necessary roughness, I used little wings of metal soldered to the pin, but lately have adopted the method described by Dr. Meriam in the *Cosmos*, of putting a spiral of wire round it, soldering with gold. Two or three breakages of the pin had previously occurred when I used the ordinary gold pin, very slightly nicked, as holdfast for the cement. The plate covering the root should be fitted closely over the edges, so that nothing but the slightest line of cement may be exposed. Where the root has not been hollowed to the edge, I think it is best to cup the top of the root, so that there may be a fair body of the oxyphosphate close to the edge all round; otherwise, if squeezed out between two flat surfaces, the thin wafer thus produced is apt to be disturbed in the trimming up, especially if the operator is at all hurried, or it may become broken up and disintegrated afterwards, leaving a distinct place for the lodgment of *debris* behind the plate and the top of the root. This method is at once very simple, and in suitable cases very satisfactory. When the tooth is ready to be inserted, the root should be thorougnly dried and ready for the reception of the oxyphosphate, not too quick-setting, a portion of which should first be put up the root and another portion round the pin and under the plate, taking care that every part is covered to some extent with it, which will ensure the surplus cement being pressed out equally all round. At the moment of writing I recall one case of a very hollow central set some six years ago, and seemingly as good to-day as then; a similar root filled with amalgam would, I imagine, have a strong tendency to necrosis.

The above are the principal methods employed in treating cases of the first class, namely, incisors and canines.

2. *Bicuspids and Molars.*—In crowning these teeth I

have done comparatively little, in fact I have not gone further back than the bicuspids, and it is to these teeth I will therefore confine my remarks. With them there comes more often than any other the question of retention of part of the tooth; thus it is of common occurrence to find the outer cusp broken off and the inner one left. This some advise retaining, fitting a canine tooth to the outer edge, soldering it to a post and filling round it with almagam, using the inner cusp as a retaining point. The retention, however, of such portions does not seem to me desirable in almost any case, the result not being so strong as in the case of complete removal, and the operation itself is generally more difficult. Unless a very large part of the cusp is left, it is considerably weakened by anchorage for the amalgam. There is also difficulty where the crown is not a large one, as in a case of a lady I had some years ago, when I set the post in the root with Weston's cement, and built up the remainder with amalgam, but it was no easy matter, on account of want of room, to pack it round the post and between the natural and artificial cusps. It happened in this, as it happens in similar cases, that the natural cusp broke off, while the artificial one remained, showing what is really the weak part in such an operation.

There can be little exception taken to the Bonwill crown, or some of the slight modifications of it, for use in the case of bicuspids. Where the bite is close, of course they are not suitable, but where there is a fair amount of room they serve the purpose well, being strong and easily adjusted. For these crowns, amalgam of a quicksetting order is the best material to employ, using either the ordinary Bonwill pins set to the roots, or what I prefer, especially in shallow crowns, the Howe screw post with nut. When the latter is used it is advisable to take the Howe dovetailed crown, which has a large opening for the reception of the nut. The root is first tapped in the centre and the post screwed in, amalgam is then packed round it over the surface of the root; then the crown is slipped over the post, and some amalgam having been packed round it, the nut is screwed up and covered with more amalgam. It is not always possible, when the bifurcation of the roots occurs not very far up, to get sufficient anchorage for the screw, and in the endeavour I went through in one case, without, however, any harm resulting, the hole being covered with a gold cylinder dipped in strong carbolic, and the screw was put in deep enough to hold quite well. In fact, the Howe screw, which has a sharp cut with shallow broad space between, holds with remarkable strength in dentine, and the short pieces cut off serve the purpose of retaining screws for contour fillings. On one occasion I found this screw nut method very useful in the case of an upper canine, which helped to support a plate. The crown was broken off and the root was exceptionally small and short, so I put in a screw post and a crown with a large opening in it, into which the nut went. It has now served the purpose well for two years.

Where there are two distinct roots, the plan of a closely-fitting cap with two platinum pins, roughened as before described, and set with oxyphosphate, is an excellent one in many cases, especially in those where the bite is close, and will not admit of an ordinary shaped crown. A gold cusp may also be built up to any height to suit the hole. Theoretically, at any rate, a band or any collar might be used in many cases of bicuspid roots, but as I have had no practical experience of their application, I forbear to say anything further in regard to it, but will leave it to those who have employed it or have seen the results of the practice. I am sure we should like to hear from our American friends, shall I call them, who may be able to speak from what they have seen of the extent of this practice, and whether many practitioners are opposed to it, as certainly some are.

It may be seen now that, as I am beginning to speak of methods I know not of, I am tending to stray beyond the limits I commenced with, so it will be wiser for me to bring these somewhat discursive remarks to a close. I only hope they may have been of some interest to you, and that you will freely give your own experience in the discussion.—*Dental Record.*

---

ARTICLE II.

### IMMEDIATE ROOT FILLING.

